# Is There Anything New in Canine AGASACA?

**DOI:** 10.3390/vetsci11120629

**Published:** 2024-12-06

**Authors:** Marzia Cino, Marina Martano

**Affiliations:** Department of Veterinary Medical Sciences, University of Parma, Strada del Taglio 10, 43126 Parma, Italy; marzia.cino@unipr.it

**Keywords:** AGASACA, apocrine glands, hypercalcemia of malignancy, lymphadenectomy, iliosacral lymph nodes, prognosis

## Abstract

Apocrine gland anal sac adenocarcinoma (AGASACA) is a locally invasive tumor with a high potential for early metastasis. The most recent studies indicate that 23.4–83% of dogs have metastases to the iliosacral lymph nodes (LNs), and 2.1–31% have distant metastases to other organs at the time of first presentation. Usually, only one anal sac is affected, but bilateral involvement has been reported in 4–20% of dogs. About 16–53% of dogs present with paraneoplastic hypercalcemia. The most recent studies show an overall improvement in median survival time (MST) (15–28 months) for dogs with different stages of AGASACA treated with surgery and adjuvant therapy compared to those treated with chemotherapy alone (6.9 to 8.7 months). The highest MST (22–32 months) was reported when radiation therapy (RT) was selected as a sole or adjunctive treatment. Several studies have been published to identify the subset of tumors with more aggressive behavior and higher metastatic potential. The main negative prognostic factors are the size of the primary tumor, metastatic regional lymphadenopathy at first presentation, the size and the number of the metastatic lymph nodes, distant metastases at the time of diagnosis, and the histological characteristics of the primary tumor. In this critical review, the latest advancements in histological diagnosis, staging, treatment, and prognosis of AGASACA are described. The aim is to provide a full overview of this neoplasm, focusing on the latest advancements on prognostic variables and treatments.

## Simple Summary

Apocrine gland anal sac adenocarcinoma (AGASACA) is a malignant tumor that originates from the apocrine glands of the anal sacs in dogs. It is characterized by its aggressive nature, often leading to local invasion and metastasis. AGASACA has a propensity for spreading to regional lymph nodes and distant organs, even in early stages. While typically affecting only one anal sac, bilateral involvement can occur in a significant number of cases. Paraneoplastic hypercalcemia is present in 16–53% of dogs at the time of diagnosis and its prognostic role is still unclear. Several factors, including tumor size, lymph node involvement, and histological features, can influence the prognosis. In this review, the latest advancements in the histological diagnosis, staging, treatment, and prognosis of AGASACA are described.

## 1. Introduction

Canine apocrine gland anal sac adenocarcinoma (AGASACA) is an uncommon malignant neoplasm arising from the glandular epithelium of the anal sac wall. The anal sacs are paired epithelial sacs located on the ventrolateral aspect of the anus (at 4 o’clock and 8 o’clock positions), between the external and internal anal sphincter muscles [1].

An old study reported that this neoplasm accounts for 2% of all skin tumors and 17% of all perianal tumors in dogs [2]. However, no recent epidemiological studies have been conducted to confirm the real prevalence of this neoplasm, which could potentially be higher.

AGASACA is most commonly reported in dogs aged 9–11 years (range = 5–15 years), although it can also be seen in younger animals [3,4,5,6,7,8,9]. In the earliest published articles, AGASACA was described as a condition affecting older bitches, with a reported prevalence of 79–100% in females [2,10,11]. Most recent studies showed no sex predisposition [3,6,7,9]. Moreover, only one study identified neutering as a risk factor, indicating a higher incidence of AGASACA in spayed compared to intact females (relative risk = 1.4) [6]. Although the study was based on recorded cases from three histopathology laboratories to minimize sample errors, other studies did not confirm these results [7,12,13]. Therefore, additional prospective and larger-scale studies are required to validate these findings.

English Cocker Spaniel (ECS) has been shown to be predisposed to AGASACA, with a mean odds ratio of 7.3 [5,6]. Additionally, British studies have provided evidence of an increased risk of AGASACA in Springer Spaniels and Cavalier King Charles Spaniels [6,14,15,16]. In a case–control study conducted on 117 ECS, the association between the major histocompatibility complex class II loci DLA-DRB1, -DQA1, and -DQB1 was investigated in 42 ECS with AGASACA and 75 controls. This study showed a significantly higher frequency of allele DLA-DBQ1*00701 in cases with AGASACA (95%, 40/44 of cases) compared with controls (73%, 55/75 of controls) [5]. No further studies have addressed the question of whether the DLA allele is responsible for the AGASACA development or if it merely indicates an indirect association with a causative locus located close by.

Other most reported affected breeds include German shepherd, Labrador, Golden retriever, Dachshund, and mixed-breed [3,6,7,13,17,18,19]. The literature shows a slight variation in the reported breeds across various studies from different geographic areas, but there is not much information available on geographic variations in the occurrence of AGASACA, except for those involving British dogs.

Many reviews on canine AGASACA have been published in textbooks and journal articles, providing information about prognostic factors, treatment, and outcome [20,21,22]. However, there are still some aspects of this tumor to be clarified. This critical review aims at providing a full overview of this neoplasm, focusing on the latest advancements in prognostic variables and treatments.

## 2. Biological Behavior

AGASACA is a locally invasive tumor with a high potential for early metastasis [13]. The most recent studies indicate that 23.4–83% of affected dogs have metastases to the iliosacral lymph nodes (LNs) [12,13,14,16,20,23] and 2.1–31% to other organs [12,14,16,20] at the time of diagnosis. These highly variable ranges could be attributed to factors such as late diagnosis, differences in imaging techniques employed for the staging, and regional LN sampling method (fine needle aspirates compared to incisional biopsy) among the studies [24].

The clinical presentation of AGASACA may be highly variable. The tumor can appear either as a visible large subcutaneous mass in the perineal region or as a small nodule, incidentally found during digital rectal examination [13,15,22]. Several studies found a significant association between the size of the tumor and the presence of regional metastases [3,12,13,20,21,25,26], but a recent study showed that 20% of the tumors smaller than 2 cm have already metastasized to the LNs at the time of diagnosis. On the other hand, 64% of the smaller tumors without metastasis at the time of initial clinical staging went on to develop progressive disease [13]. This highlights the importance of also performing a complete staging in dogs with small AGASACA. Similarly, the size of the LNs does not indicate their metastatic status, as 90% of small-/normal-sized LNs could already be metastatic [7,13,15,19].

Late-stage metastases are commonly found in the spleen (3–17.1%), liver (6–21.4%), lungs (11.4–20%), and bone (including the lumbar and sacral vertebrae, with a 7–9% incidence) [12,14,16,20]. Less commonly, metastases occur to the heart, adrenal glands, pancreas, kidneys, urinary bladder, mediastinum, omentum, retroperitoneum, and skin, as well as aortic, colic, sternal, hepatic, mesenteric, and inguinal LNs [7,14,16,20,25,26,27].

Usually, only one anal sac is affected, but bilateral involvement has been reported in 4–20% of dogs [7,18,19,28,29]. Bowlt et al. (2013) reported four cases of asynchronous AGASACA that developed from 50 to 390 days after the excision of the primary tumor, without evidence of metastatic disease at the time of the first presentation [29].

About 16–53% of dogs have paraneoplastic hypercalcemia, which may be associated with symptoms such as polyuria, polydipsia, anorexia, lethargy, or vomiting [4,7,12,13,17,18,22,26,27]. This is due to the ability of the tumor cells to synthesize the parathyroid-hormone-related polypeptide (PTH-rp), which has a similar amino acid sequence and function as the parathyroid hormone (PTH). Like PTH, PTH-rp binds to PTH receptors on osteoblasts and renal tubular cells, stimulating calcium resorption from bone and reabsorption in the kidney [11,30,31].

In a study conducted by Williams et al. (2003), although 27% of the 113 dogs with AGASACA were hypercalcemic, only 22% were reported to have polyuria and polydipsia [9]. In another study, only 65% of 17 dogs with hypercalcemia had polyuria and polydipsia [27]. Indeed, screening for the presence of hypercalcemia is indicated in all patients with AGASACA before and after therapy. Return to normocalcemia is usually seen following surgical tumor resection, and the recurrence of hypercalcemia is commonly associated with the recurrence or progression of the tumor [9,30]. The following two theories have been proposed by Meuten, in 1981, to explain why not all dogs with AGASACA develop hypercalcemia: the first is that PTH-rp is produced by well-differentiated cells, therefore, if the tumor is highly undifferentiated, it may not have functional cells to synthesize the peptide; the second explanation is that a large number of cells are required to produce enough PTH-rp to interfere with normal calcium metabolism; therefore, only larger tumors can cause hypercalcemia [11]. Studies proving the first hypothesis are lacking, and the negative prognostic role of hypercalcemia is still controversial. The second hypothesis is still debated, but a recent study demonstrated that hypercalcemia was not correlated to tumor size [7]; in contrast, in two recent studies the presence of this paraneoplastic syndrome was positively associated with the presence of LN metastases and their size; thus, the authors hypothesized a possible association between metastatic volume and the presence of hypercalcemia [14,31]. Further research could provide insights into the underlying mechanisms of hypercalcemia.

Severe and chronic hypercalcemia may result in renal damage, which may impact the quality and duration of life [14]. This could explain why it is reported that dogs with hypercalcemia have a shorter survival time than normocalcemic dogs [9,11]. Yet to date there are no studies that focus on the impact of hypercalcemia on time to documented progression (TDP), which could provide a more consistent measure of the real effects of hypercalcemia on patient outcomes [27,32].

Hypercalcemic dogs may become hypocalcemic following surgical resection of hypercalcemic AGASACA, even though this is a very rarely reported event [33,34].

Another rarely reported paraneoplastic syndrome is hypertrophic osteopathy [35,36]. Only two cases of dogs with metastatic AGASACA with ambulatory deficits, pain on palpation of the limbs, and reluctance to exercise have been described [35,36]. Interestingly, in one case hypertrophic osteopathy developed many months after the surgical removal of the AGASACA and LNs metastases, three cycles of radiotherapy, and two months of toceranib phosphate administration. The cause of the syndrome could not be ascertained, since only two small lung nodules (1 and 0.66 cm in diameter, respectively) could be found during staging, ad they were not considered as the cause of the osteopathy since their size had increased by only a few millimeters during the course of the disease (up to 2 and 1.66 cm, respectively). In addition, hypertrophic osteopathy developed 3 months after stopping toceranib phosphate administration. According to the authors, toceranib phosphate, by binding to VEGF and PDGF receptors, delayed the clinical manifestation of osteopathy, which became symptomatic after the treatment was stopped [35].

## 3. Clinical Signs and Staging

In approximately 20–57% of cases, dogs are asymptomatic, and the primary tumor may be discovered incidentally during a physical examination [9,14,18,20,23,26,37]. Therefore, the inclusion of rectal palpation as a routine part of the physical examination of any dog should be always foreseen, since it has likely increased the detection of small, asymptomatic tumors (authors’ opinion). This evaluation helps to roughly assess the size of the anal sac mass and the presence of sacral lymphadenomegaly, since the intrapelvic location of these LNs makes them difficult to detect via ultrasound [20,22,38].

Clinical signs in dogs with AGASACA can vary widely, depending on the size of the primary tumor and the presence of paraneoplastic syndromes. They include pruritus (15.8%), swelling (4.3–61%), and bleeding in the perineal area (5–24%), biting at the perineum (30%), and scooting (2–21%) [3,7,20,23,27,28]. In one study, clinical signs significantly correlated with the increasing diameter of the primary tumor [14]. Additionally, regional metastatic LNs may be enlarged, leading to abnormal stool shape (5%), stranguria (4–5%), tenesmus (14–34%), and dyschezia (19%) [7,19,20,38]. Polyuria (4.3–30%) and polydipsia (4.3–40%) may also be present and associated with hypercalcemia [18,20,23,26]. Less common signs of hypercalcemia include hindlimb weakness (9–22.5%), lethargy (4.3–13%), anorexia (17.5%), and vomiting (2–10%) [7,9,20,23]. In rare cases, dogs may exhibit pain or lameness due to bone or spinal cord metastases [7].

### 3.1. Diagnosis

Complete staging of the dogs with AGASACA should include full hematology, serum chemistry profile, ionized calcium, urinalysis, and both thoracic and abdominal imaging. In the case of severe hypercalcemia, cardiologic examination is recommended to exclude arrhythmias [38].

Even if the presence of a mass in the anal sac area is suggestive of AGASACA, fine-needle aspiration (FNA) cytology is always recommended to exclude other malignancies, such as anal sac malignant melanoma, squamous cell carcinoma or mast cell tumors, which have also been reported [39,40,41]. Fine-needle aspiration cytology of enlarged regional LNs should be performed during rectal exploration or abdominal imaging (transabdominal ultrasound-guided cytology) [38,42]. The cytological appearance of AGASACA suggests a neuroendocrine origin, and it is characterized by high cellularity, with clusters of round/polygonal cells with few characteristics of atypia, low cohesiveness and poorly defined margins. Anisokaryosis and anisocytosis may be mild and cells with free round nuclei with reticular chromatin, often indistinct nucleoli, and weakly eosinophilic cytoplasm are typically observed [43,44].

The TNM system for skin and adnexal tumors had been used to stage AGASACA [45]. However, the TNM scheme does not consider the variability of the biological behavior of AGASACA, its development in the perineal region and the proximity to important vascular and nerve structures, which may limit a wide surgical excision [4]. A new staging system was proposed by Polton and Brearley in 2007 [4]. Starting from a retrospective study on 80 dogs with AGASACA, the authors first identified significant clinical and prognostic factors, then they developed a new staging scheme validated by conducting a prospective study on 50 dogs [4]. According to this new system (Table 1), in the absence of LNs or distant metastases, the primary tumor is classified as stage 1 if it has a diameter less than 2.5 cm and as stage 2 if it is greater than 2.5 cm. When there are regional LNs but not distant metastases, regardless of the size of the primary tumor and/or if the LNs have a diameter less than 4.5 cm, the tumor is classified as substage 3a; if it is greater than 4.5 cm it is classified as substage 3b. In stage 4, metastases are spread to distant organs. Based on this staging, the median survival was greater for dogs in stages 1 and 2 (40 and 24 months, respectively) and it decreased for stage 3a and 3b, both in the retrospective (16 and 11 months, respectively) and in the prospective study (15 and 10 months, respectively). The survival time was shorter in the stage 4 group (less than 3 months). Furthermore, in this study a treatment algorithm for AGASACA was proposed based on the size of the primary tumor and any visceral metastases [4]. However, this algorithm is not universally accepted because, unlike most studies, it does not consider surgery as the treatment of choice, even in the event of recurrence, and surgical excision is not proposed based on the resectability of the tumor but on its size; moreover, chemotherapy with carboplatin is suggested for dogs in stage 3, but several studies proved no survival benefit for dogs treated with this chemotherapeutic agent [22].

In 2022, Tanis and colleagues proposed a modification of Polton and Brearley’s (2007) scheme, questioning the relevant prognostic role of LN size in the staging scheme [27]. Recent studies demonstrated that abnormal LNs should be determined based on shape, echogenicity, or contrast enhancement and that the size of both normal and pathological LNs is influenced by the size and weight of the animal [27,46]. Moreover, small LNs could be metastatic [27,46,47], and the interobserver variability in their measurement rendered this staging system challenging to replicate. In that study [27], the authors showed that the measurement of the size of the metastatic LN (mLN) was poorly reproducible, and the size of the LNs was not associated with the outcome. Conversely, the number of mLNs was significantly correlated with the outcome. In fact, dogs with four or more mLNs had significantly shorter progression-free survival (PFI = 29 days; *p* < 0.001), time to documented progression (TDP = 134 days; *p* = 0.004), and disease-specific survival (DSS = 157 days; *p* < 0.001) compared to those with less than four metastatic LNs (PFI = 110, TDP = 200 and DSS = 384) [27].

Histological analysis of all resected tissue is paramount in confirming the diagnosis of AGASACA and allowing clinicians to formulate evidence-based treatment plans with owners [14,15,22].

AGASACA are divided into three distinct histological patterns: solid, rosette, and tubular [1]. One or more of these patterns may be present in the same tumor [14,15,48,49,50].

In the solid pattern, cells are compact, surrounded by a scarce stroma, with round-oval, hyperchromatic, or normochromatic nuclei, a prominent nucleolus, and a poor eosinophilic cytoplasm [1,15,48].

In the rosette type, tumor cells, with nuclei located in the basal area with apical eosinophilic cytoplasm, are arranged radially around a small amount of eosinophilic secretion [1,48].

The tubular pattern is characterized by tubules of different diameters, bordered by cuboidal cells with hyperchromatic nuclei and abundant cytoplasm, which sometimes have apocrine vesicles near the luminal border [1,48].

Several studies have investigated the prognostic significance of histological and immunohistochemical factors [12,14,15,18,49]. The solid pattern was associated with a worse prognosis [14,15,18], with a median survival time (MST) and progression-free interval (PFI) of 471 and 377 days, respectively, compared to 949 and 1609 days for other histopathological patterns [15]. These findings were supported by another study, where dogs with solid AGASACA and metastatic regional LNs had a shorter disease-free interval (DFI) (155 days) than dogs with other histologic patterns (523 days; *p* = 0.03) [18].

The following two other histologic patterns can be found, albeit rarely: the clear cell and the signet-ring variant [1].

Since tubules, rosettes, and pseudorosettes were often observed simultaneously, recently, a new histological classification has been proposed comprising three patterns: solid, tubules/rosettes/pseudorosettes, and papillary [15]. The solid pattern shows a more malignant behavior compared to the other two patterns, according to this classification [15,18].

Other negative prognostic factors were the presence of tumor necrosis, lymphovascular invasion, peripheral infiltration, and cellular pleomorphism [14,15,18,24,49,51]. A significant association between the presence of vascular or lymphatic invasion and the presence of cells at the histologic margins has been reported (*p* = 0.001; OR 3.5 [95% CI: 1.6–7.4]). Likewise, there was a significant association between the presence of vascular or lymphatic invasion and local recurrence (*p* = 0.01; OR 2.9 [95% CI: 1.3–6.5]) [19].

Wong et al. (2021), developed a histopathological algorithm to predict survival in dogs with AGASACA. A score (0–1) was assigned to each of three parameters (tumor necrosis, histological pattern, and vascular invasion); they found that cases with a final score <1 had an MST of 906 days, significantly higher than the MST of 318 days of dogs scoring >2 [14] (Table 1). However, further prospective studies are needed to validate this algorithm.

According to Pradel et al. (2018), a mitotic count (MC) above eight was associated with a shorter disease-free interval (MC ≥ 8, 419 days, MC < 8, 760 days; *p* = 0.048) [15], but this result was not confirmed by a following study in which the same cut off was applied for statistical analysis [27].

Ki67, a nonhistone nuclear protein expressed at every stage of the cell cycle (G1-M) but not in resting cells, was not found to be useful in identifying more aggressive AGASACAs by different studies [14,18,49].

In a study conducted by Polton et al. (2007), E cadherin expression greater than 75% was associated with a higher MST (1168 days) compared to cases in which the expression was less than 75% (448 days) [51]. *Cadherins* are proteins that mediate adhesion and communication between epithelial cells and between these cells and the extracellular matrix [22]. However, this finding was not confirmed by a more recent study [49]. The authors tried to provide possible explanations for the discrepancy in the results between the two studies; in fact, both the dilutions and the isotype of the cadherins used in the immunohistochemistry procedures were different, as well as the number of cases considered (36 in the first work and 20 in the second). In addition, the dogs enrolled in the latest study were all non-metastatic stage 1 patients, while in the study by Polton et al. (2007) dogs with AGASACA at different stages, metastatic and non-metastatic, were selected [49,51].

The expression of COX-2 (cyclooxygenase isoform, whose activity increases during pathological processes) has also been demonstrated, mainly in neoplastic cells of the glandular epithelium, in both primary and metastatic lesions, thus suggesting a possible use of COX-2 inhibitors in the multimodal treatment of AGASACA. However, positivity has also been found in the ductal epithelium cells of healthy anal sacs; therefore, it is hypothesized that this enzyme plays a role in the normal functioning of the sacs [52].

Immunohistochemical analysis of AGASACA shows that 30% of tumors stain positively for one or more of the common neuroendocrine-specific proteins, including chromogranin A, neuron-specific enolase (NSE), and synaptophysin (SYP), suggesting that AGASACA may be originating from pre-existing neuroendocrine cells [48].

Positive human epidermal growth factor (HER2) staining was observed in 45% to 80% of the anal sacs affected by AGASACA and negative HER2 staining in 100% of the non-neoplastic anal sacs, suggesting a potential role in tumorigenesis of this tumor [53].

Recent advancements in the field of immunotherapy highlight the key roles of the immune checkpoint programmed death-1 (PD-1) and its ligand (PDL-1) in the immune evasion strategy of cancers. The expression of the two immune checkpoint molecules was evaluated by IHC in 41 specimens of AGASACA, showing a negative impact on survival time only in the subgroup of dogs (n = 14) treated by surgery alone [54].

### 3.2. Imaging

Considering the high rate of regional metastasis, preoperative imaging is recommended to detect both regional and distant spread, which may influence the surgical dose and prognosis. Thoracic imaging with three-view radiographs or CT scans is performed to identify lung metastases [4,40], which are rarely seen at first presentation [16,37].

Abdominal ultrasound (AUS) is the most widely used diagnostic technique to evaluate abdominal lymphadenopathy in dogs; however, although it has a greater sensitivity compared to abdominal radiography, normal sacral LNs are not easily evaluated by this technique because of their intrapelvic location [42,55]. Ultrasound can also be used to assess possible metastatic involvement of the spleen and liver, vascular infiltration, and adhesion of metastatic LNs to surrounding blood vessels, as well as to perform ultrasound-guided cytological examination of these organs [38]. To overcome the limitations of ultrasound and obtain complementary information, more sensitive diagnostic techniques, such as CT or magnetic resonance imaging (MRI), should be used, which also allow for the visualization of the extension of the primary tumor and to plan surgery [7,47,56,57,58].

A study involving six dogs histologically diagnosed with AGASACA and LN metastases demonstrated that MRI is more effective than AUS in detecting sacral lymphadenopathy [55]. MRI identified sacral lymphadenopathy in all six dogs, whereas it identified medial iliac lymphadenopathy in two cases and internal iliac lymphadenopathy in three cases. In contrast, AUS missed 67% of the lymphadenopathy detected by MRI, failing to identify any sacral lymphadenopathy and only detecting medial and internal iliac lymphadenopathy. However, AUS identified two masses which were found to be sacral LNs, which were missing with MRI images. This could be explained by the fact that the significant enlargement of these nodes allowed for the detection of their cranial-most part at the pelvic canal inlet, where ultrasound waves can penetrate [55]. The main limitation of this study was the small sample size (6 dogs), which could have biased the results.

Another study conducted on 12 dogs with AGASACA without distant metastases revealed that CT identified more LNs in the iliosacral region compared to AUS, but AUS could detect abnormal LNs that appeared normal on CT [57]. More specifically, CT identified 61 iliosacral LNs (56 normal and 5 abnormal), whereas ultrasound detected 30 iliosacral LNs (23 normal and 7 abnormal). CT found significantly more sacral (15) and medial iliac (33) LNs than AUS (which found 0 and 19, respectively). Still, there was no significant difference in the number of internal iliac LNs detected by both techniques. A limitation of this study was that only 8 out of 12 dogs underwent an excisional biopsy of at least one LN to confirm the presence of metastases. The authors of the article, therefore, did not rule out that some of the normal LNs might contain metastases [57].

These two studies demonstrate that MRI is a more sensitive diagnostic tool for detecting lymphadenopathy compared to CT and ultrasound [55,57], but both studies were conducted using a small sample size; therefore, these findings need to be confirmed. Despite this, there are no studies establishing objective and reproducible criteria for classifying metastatic LNs using advanced imaging techniques [55,56,57]. Also, in the absence of clear lymphadenomegaly, it may be difficult to determine which LNs should be sampled. Additionally, the absence of lymphadenomegaly does not necessarily rule out the presence of metastasis, and cytological examination may sometimes yield false-negative results. In fact, the reported rate of histologically metastatic but “normal-size” LNs detected through imaging was 90% (19/21) [27]. This suggests that, as for mast cell tumors [59], non-palpable/normal-size LNs could already be metastatic in AGASACA when histologically evaluated, contributing to the high rate of progressive disease observed for this tumor [55]. This also leads to questioning on the need for routine LNs excision in any case of AGASACA, regardless of the size of either the primary tumor or the LNs, or to identify LNs most likely to contain tumor metastasis. For these reasons, the use of indirect computed tomographic lymphography (ICTL) has been proposed to detect the sentinel lymph nodes (SLNs), which are the first LN(s) to which a tumor drains [56,58]. Injecting 1 mL of nonionic iodinated contrast (iopamidol) in four quadrants around the primary tumor, SLNs were identified in 92% of cases (12/13), 67% of which were ipsilateral to the primary tumor and contralateral in 33% of cases. Medial iliac LNs were the most common SLNs (33%); in 25% of cases, more than one SLN was detected [56]. This pilot study aimed to describe the feasibility of the use of preoperative mapping with CT lymphography, but data on the survival advantage with identification and excision of SLNs are lacking. The usefulness of identification of SLNs needs to be confirmed with larger studies and/or using other mapping techniques (e.g., lymphoscintigraphy or near-infrared fluorescence with indocyanine green).

A recent study involving 70 dogs with AGASACA found that the enlargement of internal iliac LNs (72.9%) was more common than that of medial iliac (62.9%) and sacral LNs (51.4%) [16]. In contrast, in another study, the medial iliac LNs were the most commonly enlarged (43.8%), followed by the internal iliac (29.2%) and sacral (14.6%) [27]. Interestingly, the side of the largest LN was ipsilateral in about 70% of cases but contralateral in 30% [27]. The researchers inferred that the enlargement of the LNs in all cases was indicative of metastatic disease, even though this statement was not always supported by a histological or cytological confirmation [16]. Moreover, the interobserver agreement in defining LN enlargement was low (56%; 95% CI: 68.5–96.6%) [16].

## 4. Treatments

The therapy of choice for the treatment of AGASACA is surgery of both the primary site and any enlarged LNs. The removal of the tumor or the induction of remission by chemotherapy and/or radiotherapy is also the main treatment for the resolution of malignant hypercalcemia [30]. However, symptomatic therapy can be administered in more severe cases, aimed at lowering blood calcium levels while waiting for the definitive treatment. Symptomatic therapy may vary according to the severity of hypercalcemia. If mild, it may be sufficient to hydrate the patient with 0.9% saline to increase glomerular filtration rate, natriuresis, and calciuresis; if moderate, once the animal has been rehydrated, furosemide (2–4 mg/kg every 8–12 h IV or PO)—a loop diuretic that inhibits calcium reabsorption at the level of the ascending loop of Henle—and, possibly, prednisone (1–2 mg/kg SID/BID PO) can be added, which rapidly reduce serum calcium levels [20,30,38]. In the case of severe hypercalcemia and clinical signs, bisphosphonates (pamidronate 1–2 mg/kg diluted in 250 mL of NaCl IV over 2 h every 2–4 weeks) can be administered [22,60].

### 4.1. Surgery

Surgical excision of both the primary anal sac mass and metastatic regional LNs is considered the mainstay of the treatment for dogs with stages 1–3 tumors [19,27]. A closed anal sacculectomy is recommended instead of the open technique to avoid tumor dissemination and contamination of the surrounding tissues [8,20,23,38,61]. Recently, a modified closed technique was described, consisting of the complete removal of the anal sac and its duct. However, the authors failed to prove that removing the duct reduced the risk of leaving cancer cells in situ [8,61]. Local recurrence rate (13–23%) was not lower than that reported for the standard closed anal sacculectomy (5–21%) [7,8,49,61]. Also, no significant difference in the complication rates between the two techniques has been reported [61]. The intraoperative and postoperative complication rates with the modified approach were reported as 4.3–8.6% and 14.3–31.9%, respectively, compared to 12.7 and 21.8% with the standard technique [8,61].

All reports on surgical complications for AGASACA are retrospective, limiting the ability to fully assess postoperative complications due to the variability in the completeness of clinical records. Major complications, such as wound dehiscence necessitating revision surgery, are more likely to be documented, while less severe issues may either go unrecorded or may not be recognized as complications. Moreover, many of the previously cited articles lack a clear definition of “complications”, and standardized grading schemes for reporting surgical complications are either not used or different among studies, limiting the comparability between the data reported [19,37,42,62].

The overall intraoperative surgical complication rate for closed anal sacculectomy is low (1.8–12.7%) [19,27,63], while postoperative complications occur more frequently (7–21.8%) [7,8,14,19,28,61,62]. According to Sterman et al. (2021), dogs that experienced intraoperative complications were 7.4 times more likely to develop postoperative complications. The most severe intraoperative issue, iatrogenic rectal perforation, was associated with a 19-fold risk of postoperative complication and a 15-fold increase in the risk of developing a surgical site infection (SSI) [19].

The most commonly reported postoperative complications in dogs undergoing AGASACA removal are listed below:

- Transient fecal incontinence (2.1–7%): it may occur when half or less of the circumference of the anal sphincter is removed or if the innervation is partially injured during surgery [19,23,62];
- Diarrhea (2%) [19];
- Incision dehiscence (5–23.4%) [23,62];
- Fistula formation (3–9%) [62,63];
- Hematochezia (5%): it is frequently observed within 8–48 h after surgery, depending on the dose of surgery applied, but it is usually self-limiting [63];
- Tenesmus (2%): generally resolves in 5 days, depending on the surgical treatment, and it may be due to the poor postoperative pain control [63];
- Surgical site infections (14–20%): usually caused by licking and dehiscence of the suture line [19,23,63].

Since bilateral anal sacculectomy may increase postoperative complication rates, unilateral anal sacculectomy is recommended for unilateral anal sac masses [20,38]. However, this suggestion has recently been debated by Franca and colleagues (2024), who reported that 20% of cases were diagnosed bilateral AGASACA, histologically but not clinically. The complication rate for the bilateral procedure of 14% (5/35), similar to unilateral sacculectomy [63].

Given the close anatomical proximity of the anal sacs to the rectum and other important structures, such as the caudal rectal nerves, wide surgical margins are not easily achievable. Unlike other cancers, the status of surgical margins (complete, close, and incomplete) does not appear to correlate with the recurrence and shorter survival time in surgically treated AGASACA patients [7,15,49]. Only one recent study reported a significant association between surgical margins (*p* = 0.008) and local recurrence [19]. Despite this, the authors recommend removing at least all visible tumor tissue to reduce the tumor burden. Recently, the use of optical coherence tomography (OCT) has been described to detect tumor cells at or within 1 mm of the gross tumor mass in five samples. However, further studies are needed to assess if this technique could be useful for the resection of this tumor, considering the absence of correlation with recurrence [64].

The presence of regional metastatic disease at the time of diagnosis has been associated with reduced MST [4,7,18,28]; nonetheless, the surgical extirpation of metastatic locoregional LNs at the time of diagnosis or the treatment of recurrent metastatic disease (e.g., by a new LNs excision) has been shown to improve survival times in affected patients [38,62,65]. In fact, a further MST of 283 days beyond a second surgical intervention following the detection of the disease recurrence was reported [62].

Lymphadenectomy is recommended in the case of evidence of metastatic regional LNs, since it appears to improve the MST and clinical signs, especially in hypercalcemic dogs and when LNs enlargement causes marked obstruction of the pelvic canal, interfering with the evacuation of feces and urine [22]. The reported complication rate associated with lymphadenectomy is relatively low (0–8.3%), with the most commonly reported complications being hemorrhage, unresectable LNs, or rupture of the LNs [7,18,62]. Other surgical complications correlated to lymphadenectomy were transient urinary incontinence (if bladder innervation is injured) and temporary hindlimb edema secondary to iliac vein occlusion [19,37]. Although the literature refers to metastatic LNs, it is not always easy to perform an FNA of all the regional LNs which appear moderately enlarged on CT or AUS imaging. However, the current recommendation is to excise all the palpable LNs in the area, since it has been proved that even smaller ones can be metastatic. Up to now a prospective study specifically dealing with this issue is still missing.

A recent study analyzed the frequency and severity of complications associated with iliosacral lymphadenectomy. Intraoperative and postoperative complications occurred in 15.7% and 10.4% of the surgical procedures, respectively. Most (56.2%) of the postoperative complications were considered mild (edema, paraparesis, hypotension, surgical site infection, and urinary incontinence). Hemorrhage was the only intraoperative complication observed, and it required blood transfusion in 45.8% of cases [37]. The maximum diameter (>4.5 cm, stage 3b) of LNs was significantly associated with an increased risk of hemorrhage and blood transfusion, while the number of LNs extirpated did not influence the risk of complications [37]. One study reported an increased complication rate in those dogs that underwent both anal sacculectomy and iliosacral lymphadenectomy in the same anesthetic procedure (n = 8/19, 42%) compared with dogs that underwent these procedures separately (n = 3/55, 5%) (*p* = <0.005) [19,28].

### 4.2. Chemotherapy

Although several chemotherapeutic agents have been evaluated over the years, including cisplatin, carboplatin, mitoxantrone, melphalan, actinomycin D, epirubicin, and chlorambucil, the use of adjuvant chemotherapy has not shown a prognostic benefit in the treatment of AGASACA [3,4,7,9,12,14,26,28,29,66,67,68,69,70]. Comparing the results of these studies is challenging due to variations in patient numbers, treatment regimens, chemotherapy protocols, inclusion criteria, follow-up, and statistical power. Also, the non-standardized use of endpoints limits the ability to compare outcomes [7,20,28] (Table 2). It has been reported that dogs treated with chemotherapy alone have the shortest MST (212 days) compared to those treated with surgery alone (500 days), radiotherapy alone (657 days), or surgery combined with chemotherapy (540 days). The longest MST was achieved in dogs treated with surgery, chemotherapy, and radiotherapy (median = 742 days) [9]. In another study, dogs receiving adjuvant chemotherapy with platinum salts had a 2.69-fold higher risk of disease progression (95% CI: 1.20–6.00) and a shorter disease-free time interval (226 days) compared to those not treated with chemotherapy (740 days) [15]. This suggests that the results may be influenced by owner willingness to pursue chemotherapy and by the fact that this therapy is often proposed in the more advanced stages of the disease (presence of LN and/or distant metastases, incomplete resection of the LNs or primary mass) [9,20,28].

In another study, the use of the same chemotherapy protocol was evaluated in 74 dogs with AGASACA [28]. Thirty (40.5%) dogs underwent surgery alone, while 44 (59.5%) dogs were treated with adjuvant carboplatin chemotherapy. Nineteen dogs (26%) underwent lymphadenectomy, and thirteen of these received postoperative chemotherapy. The protocol consisted of four IV administrations of carboplatin (300 mg/m^2^) every 3 weeks, and 40 dogs (91%) completed the protocol with minimal side effects. Although the chemotherapy protocol was well tolerated, no statistically significant differences were found between the two groups. The survival time and time to progression for dogs treated with adjuvant chemotherapy were 723 and 382 days, respectively, compared to 581 and 402 days in the surgery-alone group [28]. A similar outcome was observed in dogs treated with cytoreductive surgery and melphalan (median survival time = 20 months, range = 11.6–28.4 months) [68]. A phase I dose-finding clinical trial showed that the combination of carboplatin (200 mg/m^2^ IV every 21 days) and toceranib phosphate (2.75 mg Kg PO every other day) was well tolerated, and three of four dogs with AGASACA achieved stable disease (SD). However, the small number of cases (four) limits the statistical power; thus, this preliminary data need to be confirmed with a larger sample size [70].

In another study, dogs receiving adjuvant chemotherapy with platinum salts had a 2.69-fold higher risk of disease progression (95% CI: 1.20–6.00) and a shorter disease-free interval (226 days vs. 740 days) compared to patients treated with surgery alone. The MST was not significantly different between the two groups [15]. These data have been corroborated by other authors, reinforcing the lack of prognostic benefit of adjuvant chemotherapy [7,14]. Therefore, there is limited scientific evidence to support platinum compounds use for the control of this tumor, which contradicts the recommendations made by Polton and Brearley for stage 3a and 3b dogs [4,20,28].

Emerging research has explored the effectiveness of toceranib phosphate in treating AGASACA, showing promising results, though the number of available reports in the literature remains limited [12,26,66,69,70]. Toceranib phosphate is an oral medication approved for treating recurrent, nonresectable grades 2/3 canine mast cell tumors, with or without regional LN involvement [69]. It works by binding to tyrosine kinase receptors (TRK) on the cell surface, blocking phosphorylation and signal transduction. Toceranib also exhibits anti-angiogenic activity by inhibiting receptors involved in growth and angiogenesis (VEGFR2, PDGFR, KIT, and RET) [50,66,69,71]. In a study by Brown et al. (2012), none of the five healthy tissues from the anal sacs showed expression KIT or PDGFR-β receptors. In contrast, 2.6% of anal sacs affected by AGASACA (2/77) expressed KIT, and 19.5% (15/77) expressed PDGFR-β [50]. However, none of the tumor samples that expressed KIT also expressed PDGFR-β. Additionally, the study did not investigate mutations in these receptors [50]. Another study showed that VEGFR2 was expressed in 79% of primary tumors and 60% of metastatic tumors, while KIT was present in 33% of primary tumors and 30% of metastatic tumors. PDGFRα was expressed in all tumors, whereas PDGFRβ in only few [71].

The first evidence of the biological activity of toceranib against AGASACA came from a retrospective off-label analysis of 32 dogs with AGASACA. These dogs received a median dose of toceranib of 2.81 mg/kg orally for an average of 25 weeks. Toceranib showed a clinical benefit in 87.5% of the dogs, with 25% achieving a partial response (PR) and 62.5% experiencing stable disease (SD). The median duration of PR was 22 weeks, and SD lasted 30.5 weeks [69].

A recent study showed a high clinical benefit from toceranib (20.7% PR and 48.3% SD) in dogs with macroscopic disease. Also, the use of toceranib in dogs with microscopic settings was associated with significantly prolonged progression-free survival (median PFS = 510 days) than dogs with macroscopic disease (median PFS = 255 days) [12].

Unlike previous studies, which included dogs at different clinical stages and treatment histories, Elliot (2019) focused exclusively on stage 4 dogs with AGASACA treated with toceranib phosphate (median dose = 2.4 mg/kg) [26]. In contrast to previous studies [69], none of these dogs exhibited partial response, no one had a total response to toceranib phosphate, and 13 showed stable disease. None of the 15 dogs had to discontinue the treatment due to adverse events, but 9 were euthanized due to disease progression. Although the study lacked a control group, the reported median survival time was 356 days, higher than the 71 to 82 days reported by Polton and Brearley [4] for stage 4 dogs not treated with toceranib, leading the authors to suggest its use in dogs with distant AGASACA metastases [26].

The use of electrochemotherapy (ECT) has been evaluated in a retrospective study as the sole treatment or in combination with surgery/chemotherapy in 10 dogs with different stages of disease. Six dogs (60%) had PR, three dogs (30%) had SD, and one dog treated for microscopic disease did not show any sign of local relapse at the end of the study. Thus, the authors suggested that ECT is well tolerated and could be an alternative treatment [72].

### 4.3. Radiation Therapy

The number of studies focusing on the efficacy and toxicity of radiotherapy (RT) has increased in recent years, showing improved outcomes compared to previous reports on treatments with surgery and/or chemotherapy [17,67,73,74,75,76,77,78,79,80] (Table 3). Because of the lack of significant prognostic improvements with adjuvant chemotherapy, radiotherapy has emerged as a valid alternative for the treatment of AGASACA, especially for unresectable tumors, or as an adjuvant treatment for residual disease and regional LN metastases [17,67,73,74,75,76,77,78,79,80]. The main limitations of these cited studies are the small study population at different clinical stages of the disease and the use of other treatments that could have impacted the outcome. Also, the variation in treatment protocols across studies limits the comparison of results, leaving the optimal RT protocol unclear.

In 2003, Turek et al. described an adjuvant curative intent protocol in 15 dogs, combining 15 fractions of 3.2 Gy with mitoxantrone postoperatively [67]. The reported MST was 956 days (range = 150–1420 days), and the event-free survival (EFS) was 287 days (range = 80–1063 days), with 73% of dogs experiencing treatment failure. In addition, 100% of patients developed acute adverse events of RT [67]. Because of the high incidence of reported toxicity (33–100%) and to the density of the organs at risk (OAR) in the caudal abdomen/pelvis, irradiation with doses < 3 Gy is recommended in the pelvic region [17,67,75,77]. The most common side effects are dermatitis with dry desquamation, diarrhea, and colitis. To reduce the incidence of these effects, it has been recommended not to exceed the dose of 3 Gy per fraction in the pelvic area [75]. Unlike definitive-intent RT, in which the goal of treatment is to increase tumor-free survival, palliative hypofractionated protocols are often considered as a treatment used solely to improve quality of life, with tumor control being a secondary endpoint. Several protocols have been published [76,77,78], including a recent definitive intent moderately hypofractionated RT (12 × 3.8 Gy). In this study, the incidence of acute toxicities was similar to that previously described using definitive-intent RT protocols (73% of grade 2 and 36% of grade 3 toxicities) [75]. Meier et al. (2017) compared the outcome of dogs with stage 3 disease treated either with surgery or hypofractionated RT [77]. Interestingly, this protocol prolonged the outcome more than expected from a palliative treatment, acute adverse events were easily managed, and no late adverse events were reported. Median PFI was significantly lower for cases treated with RT (347 days), and the hazard ratio for disease progression was significantly lower with RT (HR = 0.322, *p* = 0.013) compared to dogs treated only with surgery (median PFI = 159 days) [77].

A recent study compared the outcome of 50 dogs divided into five treatment groups: RT alone (n = 22, 44%), RT with chemotherapy or targeted therapy with toceranib (n = 9, 18%), RT with toceranib (n = 9, 18%), RT with surgery (n = 5, 10%), and RT with surgery and chemotherapy or targeted therapy with toceranib (n = 5, 10%). Radiotherapy consisted of five daily fractions of 4 Gy (total dose of 20 Gy). In contrast with the results of Meier et al. (2017) [77], the MST of dogs treated with RT alone (384 days) was not significantly longer than that of the other treatment groups (RT + surgery = 450 days, RT + chemotherapy = 672 days, RT + toceranib = 628 days, and RT + surgery + chemotherapy = 703 days). The longest MST was achieved for dogs treated with surgery, RT and chemotherapy, or targeted therapy with toceranib (703 days), as previously described [9,79].

The feasibility of stereotactic RT (SRT) for the management of AGASACA has been described recently in two pilot studies [73,80]. Stereotactic RT consists of large doses of radiation delivered in a small number of fractions (3–5) to a target volume, limiting the adverse effects. The major advantage is the reduction of the treatment time and the number of anesthetic episodes. A recently published SRT study reached a median PFI of 549 days and an MST of 991 days, with mild and medically manageable side effects, but prospective studies with larger sample sizes are necessary to confirm these preliminary data [80].

## 5. Prognosis and Prognostic Factors

In the older studies, the MST of dogs surgically treated for AGASACA ranged widely, from 7.9 to 23 months, with an estimated survival rate of 65% at 1 year and 29% at 2 years [3,7,9,62,68,70]. More recent studies have demonstrated an overall improvement in MST (15–28 months) for dogs with different stages of AGASACA treated with surgery and adjuvant therapy [8,12,14,15,18,54], compared to those treated with chemotherapy alone (6.9 to 8.7 months) [3,7,9,14]. An MST of 22–32 months was reported when RT was included in the treatment plan, either as a sole or adjunctive treatment [67,79,80]. Also, dogs treated with RT showed a higher disease-free time (159–908 days) than dogs treated surgically, with or without adjuvant therapy (178–510 days) [7,12,14,17,18,49,54,67,70,75,79]. In general, there is an improvement in survival in dogs receiving any type of local treatment compared to untreated or only medically treated dogs [14]. Local recurrence varies widely from 13 to 44% and is not associated with the completeness of surgical excision [7,8,15,19,27,49,61,70]. Tumor-related death ranged from 41 to 81% [4,7,18,27,70].

Due to the variable biological behavior, several studies have been published to identify a subgroup of tumors with more aggressive behavior and higher metastatic potential. Both clinical and histopathological factors have been researched to better define the outcomes and determine the best treatment for dogs with AGASACA. However, the available data in the literature has not always been consistent, probably due to variations in patient selection, treatment with different therapies, and to the small sample size of many studies, which often limits the statistical significance of the findings. The main negative prognostic factors reported across different studies include:

- Size of the primary tumor;
- Presence of clinical symptoms;
- Metastatic regional lymphadenopathy at first presentation;
- Size of the metastatic lymph nodes;
- Presence of distant metastases at the time of diagnosis;
- Histological characteristics of the primary tumor;
- Presence of hypercalcemia.

In several studies, primary tumor size has been identified as a prognostic factor for survival, although various cut-offs have been proposed, limiting its practical application in clinical settings [4,7,9,15,18,27,76]. In one study the hypothesis that the plethora of published cut-offs were related to the different methods of measurements among studies failed to be proven [24]. In a study involving 113 dogs with AGASACA, the tumor diameter was identified as an independent predictor of survival [9]. The median tumor size was 9 cm^2^ (range 0.5–400 cm^2^); the median survival was 584 days for the 32 dogs with tumors smaller than 10 cm^2^ and 292 days for the 24 dogs with AGASACA larger than 10 cm^2^. However, dogs treated differently were included, complicating the interpretation of the results [9]. In another retrospective study, the outcome of a more uniform population of dogs with surgically resected AGASACA < 3.2 cm (equivalent to an area of 10 cm^2^), without metastases and treated with surgery alone was examined. For all 34 dogs, the median survival was 1237 days (3.4 years) and the median time to disease progression was 851 days (2.3 years) [49].

In the study by Polton and Brearley (2007), the median survival time for dogs without LN metastases and a primary tumor size < 2.5 cm was 1205 days, whereas it was 722 days for those with tumors larger than 2.5 cm [4]. In a study by Pradel et al. (2018), the size of the primary tumor was significantly associated with the presence of clinical signs and LN metastases at the time of diagnosis [15]. This mirrors the finding of another study, in which tumors > 2.5 cm were 3 to 4 times more likely to present with metastasis than tumors < 2.5 cm. The variability in this increased risk is due to the different tumor measurement techniques across studies [24]. A recent study found a statistical correlation between the size of the primary tumor (≥2 cm) and the presence of metastasis at the time of diagnosis (*p* < 0.0001) [13]. The same cut-off was found to be significantly correlated to the outcome in another study (MST < 2 cm = 678 days; MST ≥ 2 cm = 360 days; *p* > 0.048) [14]. Dogs with AGASACA < 4 cm in size showed a PFI and MST (518 and 773 days, respectively) higher than dogs with tumors larger than 4 cm (251 and 433 days, respectively). Still, a similar cut-off (4.2 cm) was not found to be significant in another study [7]. Morello et al. (2021) specifically investigated the prognostic role of tumor size in an AGASACA population with regional metastases. Dogs with AGASACA < 5.25 cm had an MST significantly longer (877 days) than dogs with AGASACA ≥ 5.25 (220.5 days), although no significant difference was found in DFI [18].

A study by Pradel et al. (2018) correlated the presence of systemic (anorexia, polyuria and polydipsia, tenesmus, and constipation) and local (clearly visible mass, excessive licking, and bleeding) clinical signs at diagnosis with PFI and MST. Dogs with systemic clinical signs had a shorter PFI (median = 142 days, 95% CI: 0–337 days) and MST (median = 293 days, 95% CI: 199–386 days) than dogs with only local clinical signs (median PFI = 430 days, MST = 773 days) or asymptomatic dogs in which the tumor was found incidentally (median PFI = 1609 days, MST = 0–2084 days) [15].

Metastatic regional lymphadenopathy at presentation is associated with a worse prognosis in most studies [7,14,15,18,27,54,67,77]. The median disease-free time for dogs with LN metastasis at diagnosis (110–283 days) is significantly shorter compared to dogs without (529–940 days) [7,15,18,70]. The median expected survival for dogs with AGASACA without metastases at diagnosis ranges from 925 to 1237 days [15,18,49]. Dogs with LN metastases at the time of surgery have a 2.5-fold higher risk of disease progression than dogs without LN metastases, as well as a 2.31-foldhigher risk of tumor-related death [7]. Although the prognosis is worse for dogs with LN metastases, their outcome improved after excision of metastatic LNs. Dogs surgically or medically treated at relapse had a median disease-specific survival (DSS) of 490 days (95% CI: 469–999), whereas dogs without treatment at relapse had a DSS of 212 days (95% CI: 186–335) [27].

According to the new staging system by Polton and Brearley (2007), LN size is statistically associated with prognosis and may address the choice of treatment. In the prospective part of their study, MST for dogs in substage 3a (i.e., with a primary tumor of any size and LNs < 4.5 cm) was 448 days (range = 386–590), while in substage 3b (i.e., with LNs > 4.5 and primary tumor of any size), it was 294 days (range = 129–459). Other cut-offs have been proposed (2.5, 1.6, and 5 cm) but, as previously mentioned, the prognostic role of LNs size has been questioned and the number of total metastatic LNs has been proposed as a better prognostic factor [27].

The presence of distant metastases is another prognostic factor. The MST for dogs with distant metastases ranges from 71 to 356 days [4,9,14,26], and it is significantly shorter than that of dogs without distant metastases (252–1237 days) [14,18,49,54].

The prognostic value of the different histological factors has been extensively described in the most recent studies [14,15,18,27]:

- Histological pattern: dogs with predominantly solid pattern tumors have shorter DFI (median = 155–377 days) and MST (median = 352–471 days) than patients with tumors characterized by other patterns (median PFI = 523–1609 days and MST = 631–949 days) [14,15,18];
- Peripheral tumoral infiltration: Defined as the presence of neoplastic aggregates located separately from the primary tumor, within the peripheral connective tissue and/or in the muscle. If marked, the reported median PFI and MST are 421 and 590 days, respectively, and it is shorter than in cases with moderate or no infiltration [15];
- Necrosis: if present, the median of PFI and MST reported are 221–378 and 282–555 days, respectively; if absent, 376–1609 and 559–834 days, respectively [14,15,18];
- Lymphovascular invasion: if present, it is significantly associated with shorter MST (142–367 days) and PFI (273–293 days) [15,18,54]. In one study, the only risk factor significantly associated with local recurrence was the presence of vascular or lymphatic invasion (*p* = 0.008; OR 3) [19].

The role of hypercalcemia is still debated and controversial. While some studies indicated its negative impact on survival time [9,11,24], in the most recent ones no statistically significant difference in survival between hyper- and normo-calcemic dogs was detected [3,7,13,14,27,28,68]. In some papers, its role is still unclear [15,18]. However, studies with a larger number of cases, enrolled at the same clinical stage and treated with the same therapeutic modalities are needed to define the real prognostic value of hypercalcemia [22].

## 6. Conclusions

AGASACA are rather rare tumors arising from canine anal sacs. They should be diagnosed at an earlier stage by always performing a digital rectal palpation on all middle-aged animals referred to a veterinary consultation, regardless of clinical signs. Once diagnosed, LN metastasis should be assessed, although the need for excision of any normal-sized SLN is still debated. The number of metastatic LNs appears to have a better prognostic value than their size, but there are no imaging techniques sufficiently reliable in determining both size and metastatic status, and only histology can confirm the diagnosis. The excision of recurrent LNs metastasis improves the survival time, as opposed to the occurrence of distant metastases. The prognostic role of hypercalcemia is still debated, as well as that of the size of the primary tumor, while the presence of distant metastasis at presentation and histologic subtypes seem to have prognostic significance. Surgical excision of both the primary tumor and the SNLs is the mainstay of treatment; adjuvant chemotherapy has not been proven to be beneficial, but targeted therapy, as well as RT, have shown promise in improving survival outcomes. Prognostic factors, including tumor size, clinical signs, and histological characteristics, play a crucial role in determining the outcome, though there is inconsistency in their application and significance across studies. Prospective studies conducted on more homogeneous and larger groups of dogs are warranted to unravel the aspects of the treatment and prognosis of this tumor that are still unclear.

## Figures and Tables

**Table 1:** Histopathological algorithm to predict survival in dogs with AGASACA proposed by Wong et al. (2021) [14].

Parameter	Group	Score
Tumor necrosis	None	0
	Present	1
Predominant histological pattern	Rosette or tubular	0
	Solid	1
Vascular invasion	None	0
	Present	1

**Table 2:** Summary of the most recent studies on chemotherapy in AGASACA. IV = intravenously; PFI = progression free interval; OST = overall survival time.

Authors	Chemotherapy Agent and Dosage	Number of Cases	Outcome	Comments
Wouda et al., 2016 [28]	Carboplatin 300 mg/m^2^ IV every 3 weeks for 4 cycles	70 Dogs 44 dogs: Surgery alone 30: Surgery + carboplatin	MST = 581 days TTP = 402 days MST = 723 days TTP = 382 days	No statistically significant difference in MST or progression between groups
London et al., 2012 [69]	Toceranib 2.81 mg/kg orally every other day	32 Dogs	87.5% of dogs had clinical benefit 62.5% SD 25% PR Median PR duration = 22 weeks Median SD duration = 30.5 weeks	Promising results, particularly for dogs with microscopic disease Potential for prolonged progression-free survival
Elliot 2019 [26]	Toceranib 2.4 mg/kg orally every other day	15 Dogs	20.7% PR 48.3% SD PFI = 354 days MST = 356 days	MST for treated dogs was substantially longer than survival times (71 vs. 82 days) previously reported for dogs with stage 4 AGASACA treated with non–tyrosine kinase inhibitor [4]
Heaton et al., 2020 [12]	Toceranib 2.7 mg/kg orally every other day	36 Dogs 80.6% Gross disease 19.4% Microscopic disease	PFS = 255 days OST = 350 days PFS = 510 days OST = 732 days	69% of dogs treated in the macroscopic disease setting experienced clinical benefit from treatment (20.7% PR, 48.3% SD)

**Table 3:** Summary of the published studies on radiation therapy in AGASACA. RT = radiotherapy; N = number; Gy = Gray; mOS = median overall survival; mEFS = median event-free survival; *3D*-CRT, 3D conformal treatment plans; NR = not reported; mPFI = median progression-free interval; AT = additional therapy; mTTP = median time to progression.

Authors	Type of Radiation Therapy	Protocol	Adjunctive Therapy	N of Cases	Acute Toxicity	Late Toxicity	Outcome
Turek et al., [67]	Adjuvant definitive-intent RT Non-graphic manual planning	15 × 3.2 Gy Total dose 48 Gy	Surgical resection of the primary tumor + mitoxantrone (5 mg m/^2^ IV every 21 days)	15	Desquamation in 14 dogs (93%): -Grade 1 = 1 dog Grade 2 = 2 dogs Grade 3 = 11 dogs Colitis in 11 dogs (73%) Tenesmus in 12 dogs (80%) Perianal discomfort in 15 dogs (100%)	Tenesmus in 4 dogs (20%) Rectal stricture in 2 dogs (13%) Chronic diarrhea in 4 dogs (20%) Fecal incontinence in 1 dog (7%)	mOS = 956 days 66% of dogs alive at 1 and 2 years mEFS = 287 days
McQuown et al. [76]	Hypofractionated RT 3D-CRT based on CT imaging vs. Non-graphic manual radiography	3–10 × 3–9 Gy Total dose 18 –32 Gy	Surgery Chemotherapy (different agents)	77	Colitis in 21 dogs (27%): Grade 1 = 18 dogs Grade = 3 dogs Alopecia and/or erythema: Grade 1 = 9 dogs (12%): Desquamation without edema: Grade 2 = 4 dogs (5%)	Stricture in 2 dogs (3%) Alopecia in 3 dogs (4%) Leukotrichia in 2 dogs (3%)	PR in 38% of dogs Clinical benefit in 63% of dogs mOS = 329 days mPFI = 289 days
Meier et al. [77]	Hypofractionated RT 3D-CRT based on CT imaging	8 × 3.8 Gy Total dose 30.4 Gy, over 2.5 weeks	15 dogs treated with surgery 13 dogs treated with RT Chemotherapy (different agents)	28 Dogs with stage 3b	Grade 0 = 8 (62%) dogs Grade 1 skin toxicity = 4 dogs (31%) Grade 2 toxicity Lower gastrointestinal tract = 1 dog (8%)	Not observed in any patient	mOS = 347 days mPFI = 159 days
Williams et al. [17]	Adjuvant definitive-intent RT 3D-CRT based on CT imaging	20 × 2.5 Gy Total dose 50 Gy	Surgical resection of the primary tumor and lymphadenectomy Chemotherapy	12	Dermatitis Grade 2 = 6 dogs (50%) Grade 3 = 6 dogs (50%) Anusitis Grade 3 = 12 dogs (100%)	Dermatitis - Grade 1 = 9 dogs (75%) Colitis Grade 2 = 2 dogs (17%)	Recurrence 8% mOS = 771 days
Swan et al. [80]	Stereotactic Body RT	Primary tumor site: 8 Gy per fraction Total dose 22–24 Gy Node site: 7–5-8 Gy per fraction Total dose 22.5–24 Gy	4 dogs: surgery + RT 5 dogs: Surgery + RT + Chemotherapy 3 dogs: RT + Chemotherapy	12	Alopecia and erythema: Grade 1 = 11 dogs (91%) Desquamation with edema: Grade 2: 1 dog (8%) Dyschezia: Grade 1 = 3 dogs (25%) Diarrhea: Grade 2: 9 dogs (75%)	Rectal stenosis: 1 dog (8%) Tenesmus: 1 dog (8%)	mOS = 991 days mPFI = 549 days
Körner et al. [75]	Definitive-intent, moderately hypofractionated RT Image-guided intensity-modulated RT	12 × 3.8 Gy Total dose 45.6 Gy	Surgery	11	Grade 2 toxicity = 8 dogs (73%) Grade 1 toxicity = 4 dogs (36%)	Not observed in any patient	mPFI = 908 days
Mendez Valenzuela et al. [79]	Hypofractioned RT	5 × 4 Gy Total dose 20 Gy	22 dogs: RT 18 dogs: RT + chemotherapy 5 dogs: RT + surgery 5 dogs: RT + surgery + chemotherapy	50	72% of all patients Grade 1 = 4 dogs (11%) Grade 2 = 30 dogs (30%) Grade 3 = 4 dogs (11%)	21% of all patients - Grade 2 intermittent diarrhea = 6 dogs (18%) - Grade 3 non-healing skin ulceration = 1 dog (3%)	mOS (RT alone) = 384 days mOS RT + AT = 628 days mPFI = 337 days

## Data Availability

No new data were created or analyzed in this study. Data sharing is not applicable to this article.
